# Cost-effectiveness of clinical breast examination screening programme among HER2-positive breast cancer patients: a modelling study

**DOI:** 10.1007/s12282-022-01398-2

**Published:** 2022-08-26

**Authors:** Tran T. Ngan, Siobhán Browne, Martha Goodwin, Hoang Van Minh, Michael Donnelly, Ciaran O’Neill

**Affiliations:** 1 Centre for Public Health, Queen’s University Belfast, Belfast, UK; 2 Centre for Population Health Sciences, Hanoi University of Public Health, Hanoi, Vietnam; 3 Roche Products Ireland Limited, Dublin, Ireland

**Keywords:** Clinical breast examination, Breast cancer, Cost-effectiveness analysis, Health economics, Low- and middle-income countries

## Abstract

**Purpose:**

For many low- and middle-income countries (LMICs), breast cancer (BC) screening based on mammography is not a viable option. Clinical breast examination (CBE) may represent a pragmatic and cost-effective alternative. This paper examines the cost-effectiveness of CBE screening programme among a patient group for whom its cost-effectiveness is likely to be least evident (HER2-positive patients) and discuss the wider implications for BC screening in LMICs.

**Methods:**

A Markov model was used to examine clinical and economic outcomes over a life-time horizon from the patient, public payer, and healthcare sector perspective. HER2-positive patients entered the model at either disease-free survival or metastatic BC state. The downstaging effect of CBE determined the starting probabilities in the no-screening and screening scenarios. The model used a monthly cycle length, with half-cycle correction. Costs and outcomes were discounted at 1.5% annually.

**Results:**

Compared with no-screening, the cost-effectiveness ratio (ICER) per quality-adjusted life-year gained for the CBE screening programme was $1801, $2381, and $4179 from three mentioned perspectives, respectively. The finding of cost-effectiveness remained robust to a range of sensitivity analyses. The parameters to which ICERs are most sensitive are average age of cohorts, reduction in proportion of metastatic patients at diagnosis, cost of CBE, and BC detection rate of the programme.

**Conclusion:**

For HER2-positive patients and compared with no-screening, CBE screening programme in Vietnam is cost-effective from all investigated perspectives. CBE is a ‘good value’ intervention and should be considered for implementation throughout Vietnam as well as in LMICs where mammography is not feasible.

**Supplementary Information:**

The online version contains supplementary material available at 10.1007/s12282-022-01398-2.

## Introduction

 The perception that cancer is a disease of high-income countries (HICs) no longer holds true particularly regarding breast cancer (BC) as low- and middle-income countries (LMICs) currently account for the largest share (53%) of new cases worldwide [1]. The low 5-year survival rate in LMICs (which varies from 13 to 50% compared with more than 80% in HICs) may in part be explained by the high proportion of late stage of cancer at diagnosis [1]. This situation underscores the importance of early detection through screening. However, for many LMICs, the mammography-based screening programmes used in HICs are unlikely to be feasible or demonstrate sufficient value for money [2, 3]. WHO urges LMICs to look at alternative low-cost screening modalities like clinical breast examination (CBE) and analyse its cost-effectiveness before implementation [4]. In Vietnam, currently, there is no BC screening programme, although BC has the highest prevalence (among female cancers), the highest number of cancer-related deaths, an increasing incidence, and a high rate of late-stage cases at diagnosis (64.2% at stage III or IV) [5]. Thus, there is a need for a cost-effectiveness analysis (CEA) of a CBE screening programme versus no-screening.

Up to now, Lan NH et al. (2013) is the only study that conducted a CEA of CBE screening programme versus no-screening in Vietnam. The study adopted a public payer perspective with cost inputs based on 2001–2006 data and outcomes expressed as life-years lost (LYS). It concluded that CBE for women aged 40 to 55 years is highly cost-effective [6]. However, costs from this study were based on outdated data and provided poor estimates for the current situation due to advances in treatment such as the introduction of targeted therapy for HER2-positive patients. Besides, with the recent movement towards patient-centred care, measuring outcomes in terms of health-related quality of life (HRQoL) rather than just LYS and performing the CEA from the patient perspective are increasingly important [7], especially for Vietnam where patient out-of-pocket payment (OOP) accounted for approximately 45% of the total health expenditure in 2017 [8]. In this context, patients’ OOP in Vietnam should not be overlooked and assessing cost-effectiveness from other perspectives is needed.

The downstaging effect of CBE would be expected to increase survival and increase/reduce costs depending on BC subtype. The costs of treatment for general BC patients in Vietnam were 66% and 148% higher if they were diagnosed at stage II and III, respectively, compared with stage 0/I [5]. In contrast, the costs of treatment for HER2-positive patients would be expected to increase as patients diagnosed at earlier stage of BC (stage I–III) tend to opt for targeted therapy, while patients diagnosed with metastatic BC (stage IV) chose chemotherapy only to save cost. This choice pattern is a consequence of the remarkably higher cost of targeted therapy in Vietnam which was 10 times more expensive than the total cost of all other treatments combined due to its high cost in nature and low coverage from health insurance (48% for Trastuzumab and 0% for Pertuzumab) [5]. However, if CBE screening is shown to be cost-effective among HER2-positive patients for whom a rise in treatment cost related to earlier detection is likely to be greatest, it is highly likely that it will be even more cost-effective for all BC patients. In this context, the fascinating question is how cost-effective CBE screening programme is among HER2-positive patients.

This paper presents the results from a CEA of a CBE screening programme versus no-screening for the group of HER2-positive patients. The study adopts not only a public payer perspective but also a patient perspective and an overall healthcare (patient plus public payer) perspective for the analyses.

## Methods

Breast cancer progression from the point it is diagnosed to when treatment is performed and afterwards until the death was modelled using a multi-state Markov model, constructed in Microsoft Excel. To increase the comparability of results, we used the model published by Younis T et al. (2020) which resembles the structures published by other authors and accepted by several health technology assessment agencies [9].

### Model structure

**Fig. 1:**
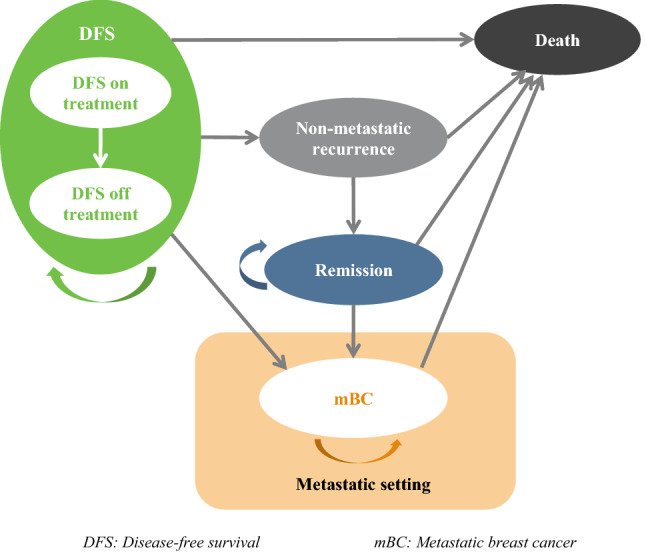
Model of breast cancer progression from diagnosis, to and after treatment

Figure 1 provides a schematic view of the model structure and the transitions between six health states: (1) Disease-free survival (DFS) on-treatment, (2) DFS off-treatment, (3) Non-metastatic recurrence (i.e., local/regional recurrence), (4) Remission, (5) Metastatic breast cancer-mBC, and (6) Death. Patients entered the model in either the DFS or mBC health state depending on their initial diagnosis at stage I/II/III or IV, respectively. Patients can remain in DFS off-treatment, remission, or mBC state as long as they did not experience recurrence or death. Patients in the DFS on-treatment state and non-metastatic recurrent are assumed to receive either targeted therapy or chemotherapy that lasts maximum 12 months (= standard length of treatment for targeted therapy), meaning that patients can only remain in these health states for that maximum length of time.

The model uses a monthly cycle length, with half-cycle correction applied, to account for mid-cycle transition. The starting age of patients in the model is 44 years which was the mean age at diagnosis of HER2-positive patients in Vietnam. A discount rate of 1.5% was applied to both costs and outcomes [10]. Other discount rates (0–3.5%) were used in sensitivity analyses.

### Parameters and data resources

#### Starting and transition probabilities

Inputs for starting and transition probabilities were derived from the literature. The starting probabilities describe the initial distribution of the patients into DFS and mBC health states, and were deemed to differ between screening and no-screening scenario due to the downstaging effect of CBE. Starting probabilities in no-screening scenario were obtained from a BC epidemiological study among Vietnamese women in the period of 2001–2007 [11]. Starting probabilities in CBE screening scenario were derived from a pilot CBE screening programme run in eight provinces of Vietnam in 2008–2010 [12].

The probability of remaining in the DFS health state has been estimated based on a parametric function that was fitted to the Kaplan–Meier DFS curves derived from patient-level data of the HERceptin Adjuvant (HERA) trial (data were supplied in personal communication with Roche—funder of the HERA trial). If not remaining, patients can experience recurrence or death. Apart from DFS curves, the transition probabilities from DFS to recurrence were further adjusted by the cure threshold, maximum cure rate, and the split between non-metastatic recurrence and mBC [13].

The probability of moving from non-metastatic recurrence to remission was based on treatment guidelines. Due to the absence of data in Vietnam, the probability of moving from remission to mBC was based on a Canadian study of 12,836 BC patients [14].

Mortality probabilities of patients in DFS, non-metastatic recurrence, and remission were based on the background mortality derived from Vietnamese life tables [15] (In HERA trial, the risk of dying for patients in these health states was lower than the background mortality). The mortality probability of mBC HER2-positive patients was assumed to be the same with that of general BC patients reported in the study of Lan NH et al. (2013) [16].

#### Costs and utilities’ inputs

Inputs for costs (from patient perspective) and utilities were derived from a sub-sample of only HER2-positive patients/survivors in a study on HRQoL of Vietnamese BC patients/survivors and cost of treatment. Details of these studies methodology and results on HRQol and cost of treatment among general BC patients/survivors (full sample) are available elsewhere [5, 17]. Cost inputs for health services and drugs from public and the payer perspective were obtained from public health service price list regulated by Ministry of Health [18] and National Cancer Hospital (data were supplied in personal communication), respectively.

The model was run with three scenarios of cost inputs: (i) patient perspective (i.e., out-of-pocket payment), (ii) public payer perspective (i.e., social health insurance), and (iii) healthcare sector perspective [i.e., the sum of (i) and (ii)]. Due to the absence of data, only direct medical costs were included (i.e., direct non-medical costs and indirect costs were excluded). The costs incurred by patients and by public payer were mutually exclusive. All costs were adjusted to 2019 price year using a Gross Domestic Product deflator index for Vietnam and are presented in Vietnamese Dong (VND) and US dollars ($).

The model outcomes were expected quality-adjusted life-years (QALYs). Utilities of non-metastatic recurrence and remission health states were assumed to equal those of DFS on-treatment and DFS off-treatment, respectively.

### Cost-effectiveness analysis

The same simulated population was modelled in CBE screening and no-screening scenarios. The incremental cost-effectiveness ratio (ICER) was calculated by dividing the difference in costs by the difference in QALYs between the two scenarios. According to WHO, interventions with ICERs less than 1–3 times gross domestic product (GDP) per capita are considered as highly cost-effective or cost-effective, respectively [19]. The respective thresholds for Vietnam were 63.2 million VND (~ $2776) and 189.6 million VND (~ $8331) [20].

### Sensitivity analyses

In deterministic sensitivity analysis (DSA), the parameters were manually changed and the impact of the change on results was analysed. As recommended by Gray et al. (2011), the range of variation for parameters derived from primary data or previous studies was based on the 95% confidence interval of such parameters [21]. Where the dispersion of the parameter was not available from data source, the range of variation would be ± 20% of the base-case [21]. In probabilistic sensitivity analysis (PSA), the choice of distributions for parameters followed the recommendation by Briggs et al. (2006) [22]. The DFS curves were described by multivariate normal distributions. Transition probabilities and utility values were modelled using beta distributions. Costs were modelled using a log-normal distribution. PSA was carried out by randomly selecting values from the chosen distributions for each parameter using Monte Carlo simulation. The number of iterations was 2000. Results from these iterations are presented in cost-effectiveness plane (C–E plane) and cost-effectiveness acceptability curve (CEAC).

## Results

Starting and transition probabilities used as input parameters in the model are presented in Table 1. The starting probabilities for DFS state were 0.833 in no-screening scenario [11] and 0.924 in screening scenario [12]. Kaplan–Meier curves illustrating the probability of remaining in DFS health state overtime are shown in Supplement Information, Fig S1. The split between non-metastatic recurrence and mBC was based on the split seen in HERA trial in which 72% of the recurrences were mBC [13]. As in this trial, the risk of progression event is decreasing towards 0 at 10 years [13], a cure threshold at month 120th and a maximum cure rate of 95% were considered in the base-case analysis. As such, at month 120th, 95% of the cohorts are deemed cured and can no longer experience a recurrence. Cost inputs for the model, by three perspectives, are presented in Table 2, while utility inputs for all health states are presented in Table 3.

**Table 1:** Starting and transition probabilities used as input parameters in the model

Parameter	Input value	Source	Sensitivity analysis
Starting probabilities for DFS state			
No-screening scenario	0.833	Duc et al. 2009 [11] (Vietnam data)	0.724–0.874
Screening scenario	0.924	Hung et al. 2012 [12] (Vietnam data)	NA
Transition probabilities from DFS			
Probability of remaining in DFS	DFS curves	HERA trial*	DFS curves in Aphinity and Katherine trials*
DFS recurrences that were mBC**	0.72	Cameron et al. 2017 [13] (HERA trial, international data)	0.576–0.864
Cure threshold (maximum cure rate reached at month)**	120		96–180
Maximum cure rate**	0.95	Assumption	0–100
DFS to death	Background mortality	WHO 2019 (Vietnamese life tables) [15]	NA
Transition probabilities from non-metastatic recurrence/remission			
Non-metastatic to remission	Automatic after 12 months if alive	Assumption	NA
Remission to mBC	0.0076	Hamilton et al. 2015 [14] (Canada data)	0.0061–0.0091
Non-metastatic or remission to death	Background mortality	WHO 2019 (Vietnamese life tables) [15]	NA
Transition probabilities from mBC			
mBC to death, year 1	0.0324	Lan et al. 2013 [16] (Vietnam data)	Pooled
mBC to death, year 2	0.0446		
mBC to death, year 3	0.0492		
mBC to death, year 4	0.0511		
mBC to death, year 5 +	0.0541		

*DFS* Disease-free survival (patients were entered the model at stage I, II, or III); *HERA* trial: HERceptin Adjuvant trial; *NA* Not applicable; *mBC* metastatic breast cancer (stage IV breast cancer); *WHO* World Health Organization

Range used in sensitivity analysis was based on the 95% confidence interval of parameters (if available) or ± 20% of the base-case

^*^Data were supplied in personal communication with Roche–funder of the HERA trial

^**^Assumptions/inputs were used to further adjusted the transition probabilities of remaining in DFS/moving to recurrence (either non-metastatic or mBC)

**Table 2:** Cost inputs for the model, by perspectives

Unit: million Vietnamese dong				
Parameter	Input by perspective (base-case) (1) Patient (2) Public payer (3) Healthcare sector			Sensitivity analysis (± 20%)
	(1)	(2)	(3)	(2)
Screening cost (one-off cost)				
Cost of CBE per person	0.0066	0.0264	0.033	0.02–0.03
Cost of CBE per diagnosis (= cost of CBE per person/the proportion of diagnostic positive patients from CBE. The latter was estimated at 0.06% [12])	11	44	55	33–55
Diagnosis cost (one-off cost)	2.76	0.79	3.55	0.59–0.98
Treatment cost for DFS on-treatment or non-metastatic recurrence				
Monthly treatment cost – those who received chemotherapy only	9.14	5.67	14.8	4.3–7.1
Monthly treatment cost – those who received targeted therapy (e.g., Trastuzumab + chemotherapy)	50.3	34.4	84.7	28.6–43.0
Monthly follow-up care cost for DFS off-treatment or remission	0.58	0.14	0.72	0.11–0.18
Treatment cost for mBC				
Monthly targeted treatment cost for mBC patients (= cost of full course of targeted therapy/average time in mBC. The former typically lasts 12 months. The latter was estimated at 40 months)	15.1	10.3	25.4	8.2—12.4
Monthly supportive care cost for mBC patients (if not receive targeted therapy or after targeted therapy)	1.23	0.24	1.47	0.18–0.30

(1) Cost inputs of diagnosis and treatment for DFS on-treatment/non-metastatic recurrence/mBC are from primary data. Cost inputs of follow-up care for DFS off-treatment/remission and supportive care for mBC were estimated from the primary data and Lan NH et al. 2013 [6]

(2) Cost inputs for health services were obtained from public health service price list regulated by Ministry of Health [18]. Cost inputs for drugs were obtained from National Cancer Hospital. Cost inputs of follow-up care for DFS off-treatment/remission and supportive care for mBC were obtained from Lan NH et al. 2013 [6]

(3) Cost inputs are the sum of costs incurred by patients and public payer ((1) + (2) = (3))

*CBE* Clinical breast examination; *DFS* Disease-free survival; *mBC* Metastatic breast cancer

**Table 3:** Utilities for health states

Health state	Mean	Mean, by age groups				Source	Sensitivity analysis
		< 40	40–49	50–59	60 +		
DFS on-treatment or non-metastatic recurrence	0.7662	0.8182	0.7922	0.6900	0.6946	Primary data, 2019 (Vietnam data)	Pooled (not adjusted by age)
DFS off-treatment or remission	0.8545	0.9229	0.8663	0.8647	0.7729		
Metastatic breast cancer (mBC)	0.6813	0.7275	0.7044	0.6135	0.6176	Younis et al. 2020 [9] (Canadian data), adjusted proportionally based on primary data of Vietnam	

*DFS* disease-free survival; *HRQoL* health-related quality of life

Table 4 shows the base-case results from the patient, public payer, and healthcare sector perspective. In all three perspectives, CBE screening programme brought about 1.1 QALYs and 1.3 life-years gained per person. The cost per QALYs gained was 41 million VND (~ $1801), 54.2 million VND (~ $2381), and 95.1 million VND (~ $4179), respectively, which suggests that from all perspectives, CBE screening programme would be considered as cost-effective.

**Table 4:** Base-case results for cost-effectiveness analysis of screening programme based on clinical breast examination

Unit for cost: million Vietnamese dong					
	Life-years per person	QALYs per person	Total cost per person (in million VND)	ICER	
				Cost per life-year gained	Cost per QALY gained
Patient’s perspective					
CBE	14.5	12.5	511.9		
No CBE	13.2	11.4	465.7		
CBE vs no CBE	1.3	1.1	46.2	36.1	41.0
Public payer’s perspective					
CBE	14.5	12.5	325.6		
No CBE	13.2	11.4	264.7		
CBE vs no CBE	1.3	1.1	61.1	47.7	54.2
Healthcare sector perspective					
CBE	14.5	12.5	837.8		
No CBE	13.2	11.4	730.5		
CBE vs no CBE	1.3	1.1	107.3	83.9	95.1
Threshold for highly cost-effectiveness					63.2
Threshold for cost-effectiveness					189.6

*CBE* clinical breast examination; ICER incremental cost-effectiveness ratio; *QALYs* quality-adjusted life-year; *VND* Vietnamese dong (currency exchange rate in September 2021: $1 ~ 22,759 VND*)*

Each plot in the C–E plane (Supplementary Information, Fig S2) represents the result from one iteration of the Monte Carlo simulation, and shows the cost and effect difference between CBE screening and no-screening. All observations were in the northeast quadrant of the C–E plane, indicating that CBE programme has significantly higher costs and effects than no-screening. All observations were also within the cost-effectiveness threshold for intervention in Vietnam.

The curves in the CEACs for CBE screening versus no-screening (Supplementary Information, Fig S3) show the likelihood that CBE screening programme is cost-effective at various willingness to pay (WTP) thresholds. At the threshold of 63.2 million VND (~ $2776 = Vietnam GDP per capita), the CBE screening programme has 100% and 70% probability of being highly cost-effective from the patient and public payer perspective, respectively. From the healthcare sector perspective, the threshold needs to be 1.5 and 2.3 times higher for the CBE screening programme has reached 40% and 100% probability of being cost-effective, respectively.

**Fig. 2:**
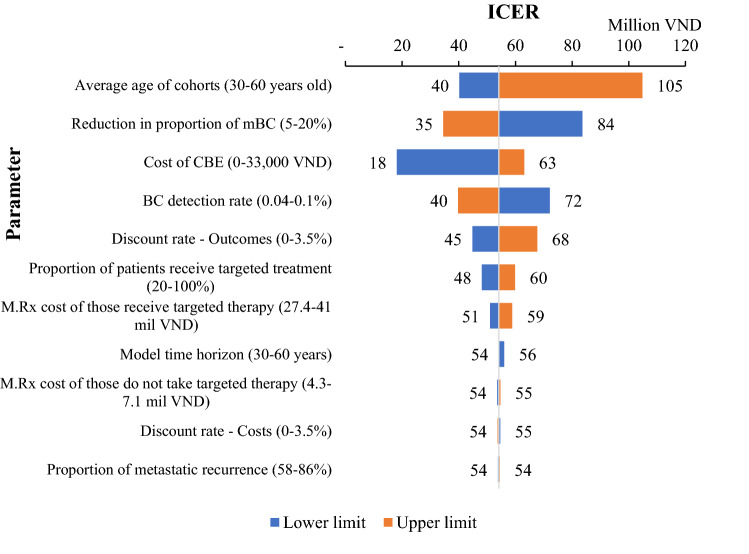
Results of deterministic sensitivity analysis, from public payer perspective

The tornado diagrams in Fig. 2 present the DSA results from public payer perspective (results for other perspective are in Supplementary Information, Fig S4). The parameters to which ICERs are most sensitive are the average age of cohorts (or average age at diagnosis), reduction in proportion of women with mBC at diagnosis (which reflects the downstaging effect of CBE), cost of CBE, and BC detection rate of a CBE screening programme. Decreasing the reduction in proportion of mBC from 9.1% to 5% would increase ICER from 54.2 million VND to 84 million VND (~ $2381 to $3691). The ICER would increase to 72 million VND (~ $3164) if BC detection rate were reduced from 0.06% to 0.04%. When all costs of CBE were covered by public payer (coverage = 80% in base-case), the ICER would increase to 63 million VND (~ $2768). All increased ICERs were still lower than the cost-effectiveness threshold.

Changing the discount rate for costs (0–3.5%), model time horizon (30–60 years), the monthly treatment cost of those who did not receive targeted therapy, and the proportion of metastatic recurrence (58–86% among total recurrence) did not significantly impact the ICER value.

## Discussion

A mammography-based screening for breast cancer is not economically feasible in many LMICs including Vietnam. The base-case analysis in this study has shown that a CBE-based screening programme would be cost-effective among HER2-positive patients for whom it would be most challenging to demonstrate cost-effectiveness. Screening increased the incremental QALYs per person. The downstaging effect of CBE resulted in the reduction of women in the mBC health state which was associated with the lowest utility value. Early stage BC also has better prognosis and longer survival time which is, in turn, associated with higher utility value (survivors have significant higher HRQoL compared with patients on-treatment [17]).

The implementation of CBE screening programme would also increase the cost incurred by both women and public payer. First, an increase in the number of patients diagnosed with stage I/II/III who are more likely to opt to targeted therapy compared to mBC patients (stage IV) significantly pushed the treatment cost higher, especially when cost of targeted therapy alone in Vietnam was 10 times higher than the total cost of all other treatments combined [5]. Patients with non-metastatic BC also faced recurrence along with cost of another treatment course; thus, the increase in number of these patients resulted in higher total cost in CBE screening scenario compared with no-screening scenario. Second, the cost of implementing the programme itself is an additional expense. Cost of CBE per person in Vietnam is remarkably cheap if compared with the cost of treatment. However, as BC detection rate from CBE screening programme (one was piloted in 2008–2010) was reported at just 0.06% [12], the cost per diagnosis was 1600 times higher than the cost of CBE per person. Thus, the detection rate plays an important role on the cost-effectiveness of the screening programme, as depicted in the DSA. This underscores the importance of efforts to retain a high detection rate including the training for doctors who perform CBE and perhaps targeting high-risk groups based on age for example.

The increase in both cost and effectiveness of CBE screening programme versus no-screening scenario yielded ICERs per QALY gained that suggest the programme is highly cost-effective from the patient and public payer perspective and cost-effective from the healthcare sector perspective. This is similar with the conclusion from studies in LMICs such as Ghana, India, and Peru where CBE screening was compared with no-screening scenario [23–25]. It should be noted that this result is highly context sensitive, and the comparator plays an important role. Studies in Japan—an HIC with a long-term, nation-wide population-based screening programme using CBE (1987–2003)—reported that CBE was not cost-effective if compared with mammography-based screening programme [26, 27]. These results, again, imply that a CBE screening programme would be cost-effective in countries where a screening programme has not been implemented yet and mammography is not feasible.

Results from PSA were consistent as all iterations of Monte Carlo simulations yielded ICERs that were lower than the cost-effectiveness threshold. The CEAC revealed that at threshold equalled to three times Vietnam GDP per capita, CBE screening programme has 100% probability of being cost-effectiveness in all perspectives. At threshold equalled to GDP per capita, it has 80 to 100% probability of being highly cost-effective from public payer and patient perspective, respectively.

Results from DSA showed that average age of cohorts (or average age at diagnosis), reduction in proportion of women with mBC at diagnosis (which reflects the downstaging effect of CBE), BC detection rate of a CBE screening programme, and the cost of CBE per person are the factors with biggest impacts on the cost-effectiveness of CBE screening programme. Of these, the average age at diagnosis of BC is a non-interventional factor dependent on epidemiological characteristics of BC in Vietnam. All other factors are related to the effectiveness and implementation of CBE screening programme. Efforts to alter these factors appropriately can contribute to increase the programme cost-effectiveness. Cost of CBE was regulated by Ministry of Health as in the category of physical examination in general which is unlikely to be changed for the sake of one programme. Reduction in proportion of mBC is linked with the detection rate as in more BC cases detected at earlier stage by screening may help decrease the proportion of mBC at diagnosis. This, once again, emphasises the necessity of improving detection rate through standardised screening process, continuous training, higher throughput, better follow-up with referral for further tests in suspicious cases, and perhaps targeting high-risk groups.

This is the first study in Vietnam that performed cost-effectiveness analysis of CBE screening programme compared with no-screening among HER2-positive patients. It is within a context of an immense burden in terms of financial and human cost of BC in Vietnam. Considering CBE screening programme is cost-effective for this particular vulnerable group, it is likely to be even more cost-effective for general BC patients among whom the potential for savings in treatment cost are even greater. Nevertheless, further research to confirm this prediction is still needed. The study is also the first to provide CEA from several perspectives including patients, public payers, and healthcare sector perspective; thus, it provides a more comprehensive picture on the cost-effectiveness of CBE screening programme in Vietnam. Most of the data inputs for the model were primary data which is recent and specific to Vietnamese patients to avoid one of the main criticisms for cost-effectiveness study from LMICs (i.e., poor quality due to the inappropriate estimates of cost and effectiveness based on data from HICs).

Despite its strength, the study has some limitations that should be considered when interpreting the findings. First, we have been compelled by the paucity of existing data on transition probabilities to group stages I–III BC to just one-state ‘early BC’ (eBC). While this follows practice adopted in the literature [9], inevitably, it will conceal heterogeneity in cost and outcomes between stages. However, among HER2-positive patients, it is noted that heterogeneity in treatment costs among eBC patients will be significantly lower than among BC patients generally by virtue of their eligibility for chemo/targeted therapy which is a key component of treatment costs. Moreover, it is noted that there is no significant difference in HRQoL among HER2-positive patients diagnosed at different stage of BC. These suggests that the forced abstraction is unlikely to be material. Second, due to absence of data, no direct non-medical costs and indirect costs were included. An analysis from societal perspective would provide a more complete view on cost-effectiveness of CBE screening programme in Vietnam. If as seems probable further savings can be achieved through avoided productivity losses for example, CBE may be even more cost-effective than suggested here. Third, the detection rate of CBE screening programme used in this CEA was from 2008 when the incidence of BC in Vietnam was around 13.8/100,000 women. More recent evidence indicates that the incidence rate was 34.2/100,000 women in 2020 [28]; *ceteris paribus*, this suggests that the detection rate should be higher than that modelled here which, in turn, will reduce ICER and make the programme more cost-effectiveness.

## Conclusions

 This analysis was not to assess the cost-effectiveness of CBE screening programme targeted only at HER2-positive women but rather to look at how cost-effective CBE screening programme is among this particular group mindful of the likelihood that among them it was least likely to be cost-effective.

For HER2-positive patients and compared with no-screening, the implementation of CBE screening programme in Vietnam is cost-effective from the healthcare sector perspective and highly cost-effective from the patient and public payer perspective. As HER2-positive patients have poorer prognosis and bear extensively higher cost of treatment for BC, it likely that a CBE screening programme would be highly cost-effective or even dominant for general BC patients. Given the results from conducting this CEA and the fact that it is infeasible to provide and deliver mammography, CBE is a ‘good value’ intervention and should be considered for implementation throughout Vietnam.

## Supplementary Information

Below is the link to the electronic supplementary material.

Supplementary file1 (PDF 404 KB)

## Data Availability

All data generated or analysed during this study are included in this published article and supplementary materials.
