# Supplementary material for: Cost-effectiveness of clinical breast examination screening programme among HER2-positive breast cancer patients: a modelling study
Source: Breast Cancer. 2022 Aug 26;30(1):68–76. doi: 10.1007/s12282-022-01398-2 (PMC9813013; doi:10.1007/s12282-022-01398-2)

Supplementary information

Fig S1 Curves illustrating the probability of remaining disease-free over time

The probability of remaining in disease-free survival (DFS) health state has been estimated based on a parametric function that was fitted to the Kaplan-Meier (KM) DFS estimates from the HERceptin Adjuvant (HERA) trial trial (data was supplied in personal communication with Roche - funder of the HERA trial). The length of follow-up in HERA trial was 11 years and as a lifetime horizon was applied in the model, it is necessary to extrapolate the data beyond the clinical follow-up period of the trial. Extrapolation of DFS curves (Figure below) was performed by fitting a parametric distribution to the observed DFS times from the study period of the HERA trial. A Gamma function was used to extrapolate the data based on the best-fit Akaike information criterion (AIC). The DFS curve used in the model is the weighted average of the two arms of the HERA trial (patients receiving chemotherapy + targeted therapy vs only chemotherapy).

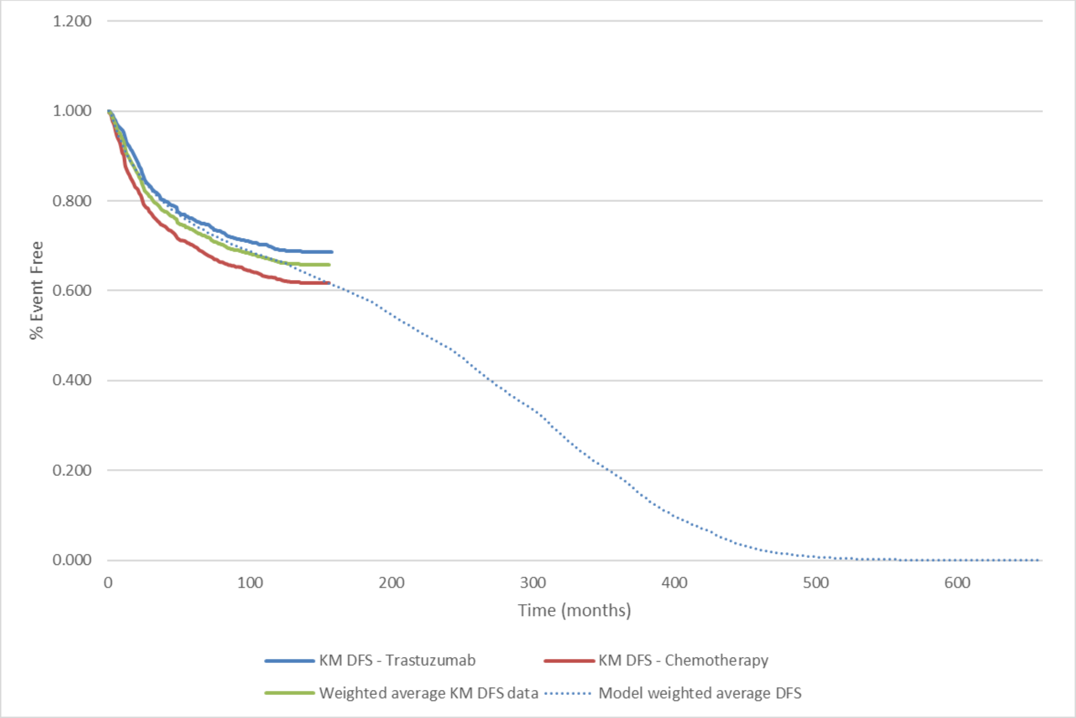

Fig S2 Cost-effectiveness planes for clinical breast examination-based screening versus no screening, from different perspectives

a. Patient perspective

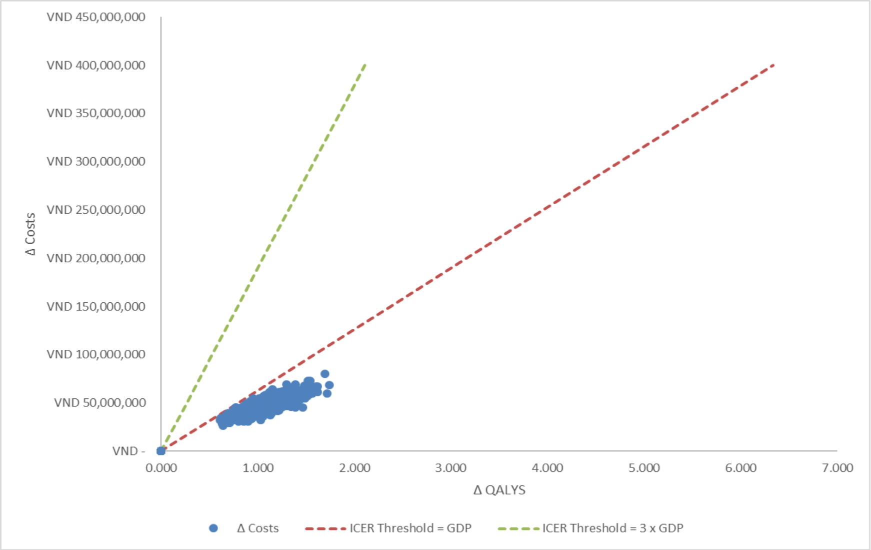

b. Public payer perspective

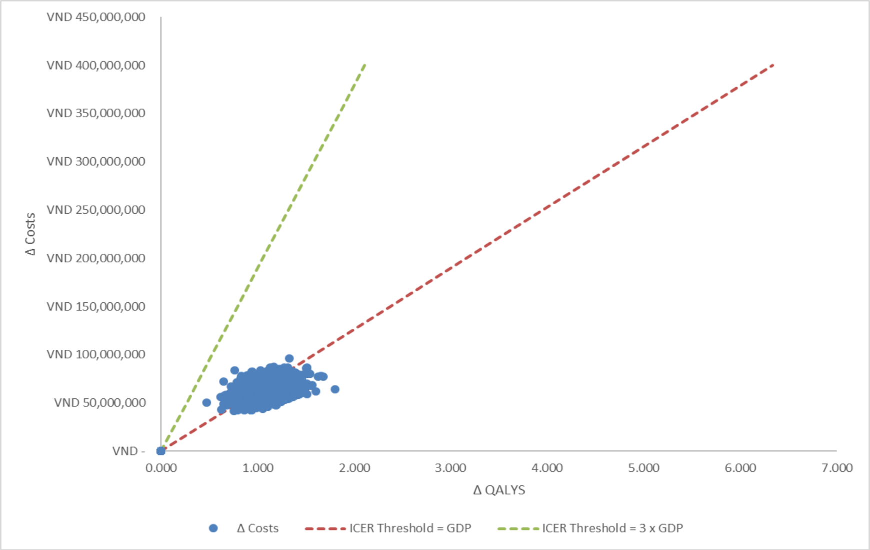

c. Healthcare sector perspective

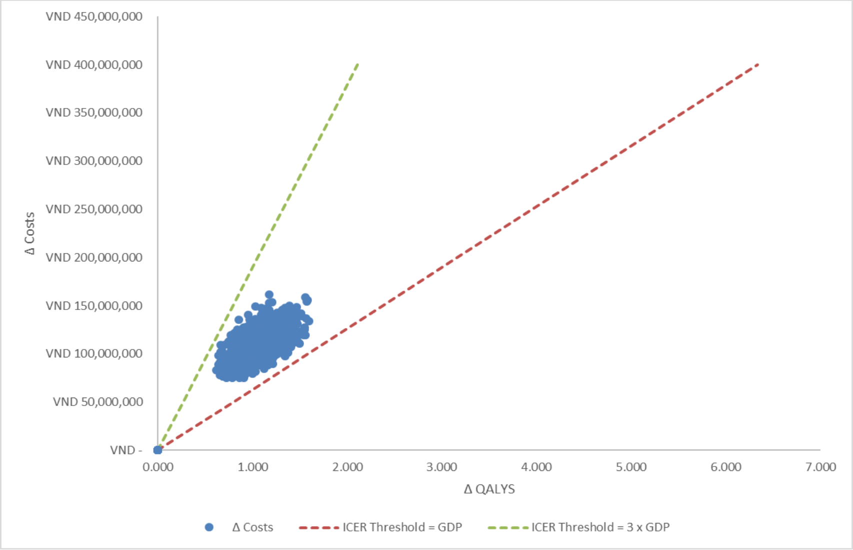

Fig S3 Cost-effectiveness acceptability curves (CEAC) for clinical breast examination-based screening versus no screening (CBE vs no CBE), from different perspectives

a. Patient perspective

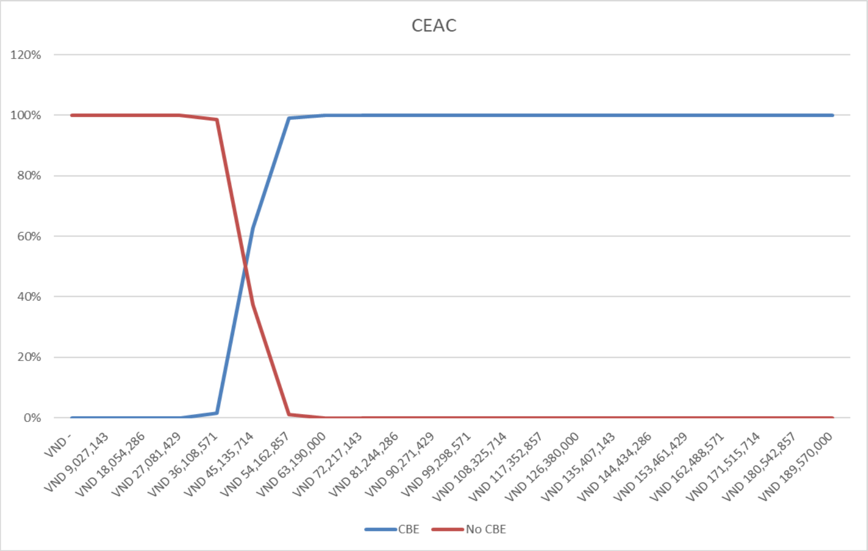

b. Public payer perspective

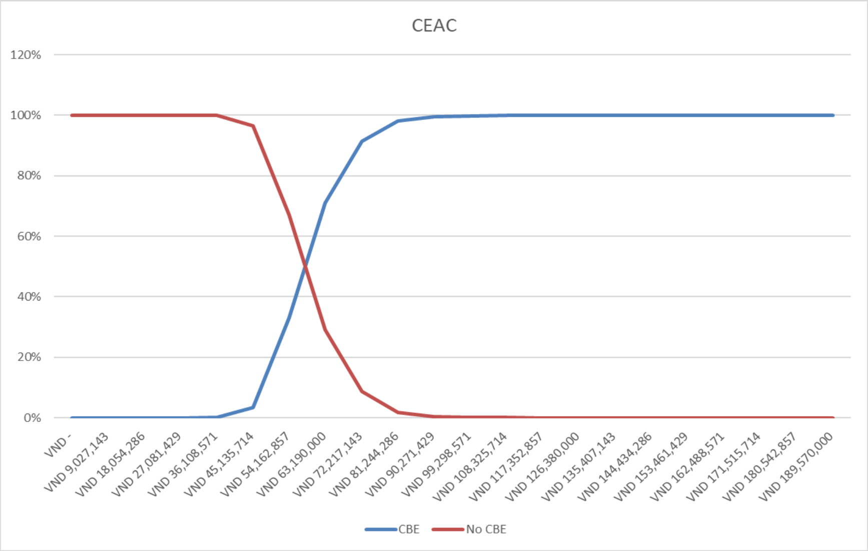

c. Healthcare sector perspective

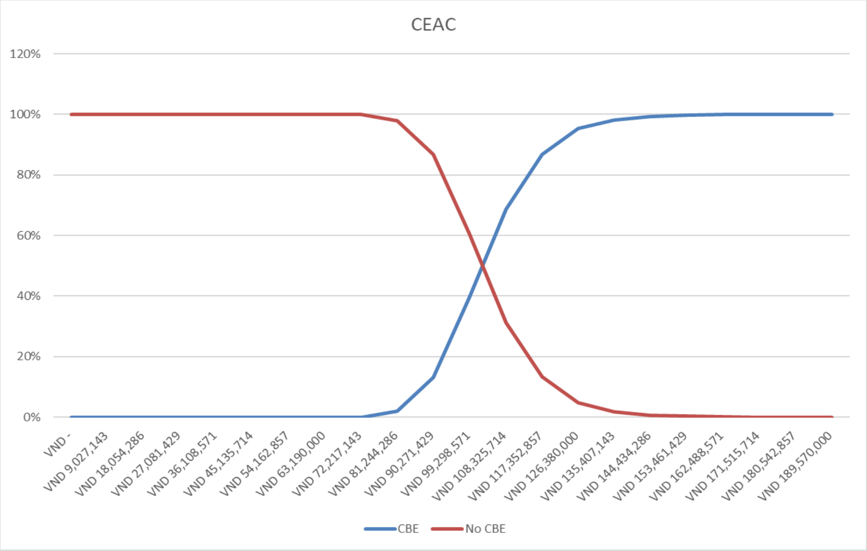

Fig S4 Results of univariate sensitivity analyses showing impact of key factors on ICERS for CBE screening in Vietnam, from different perspectives

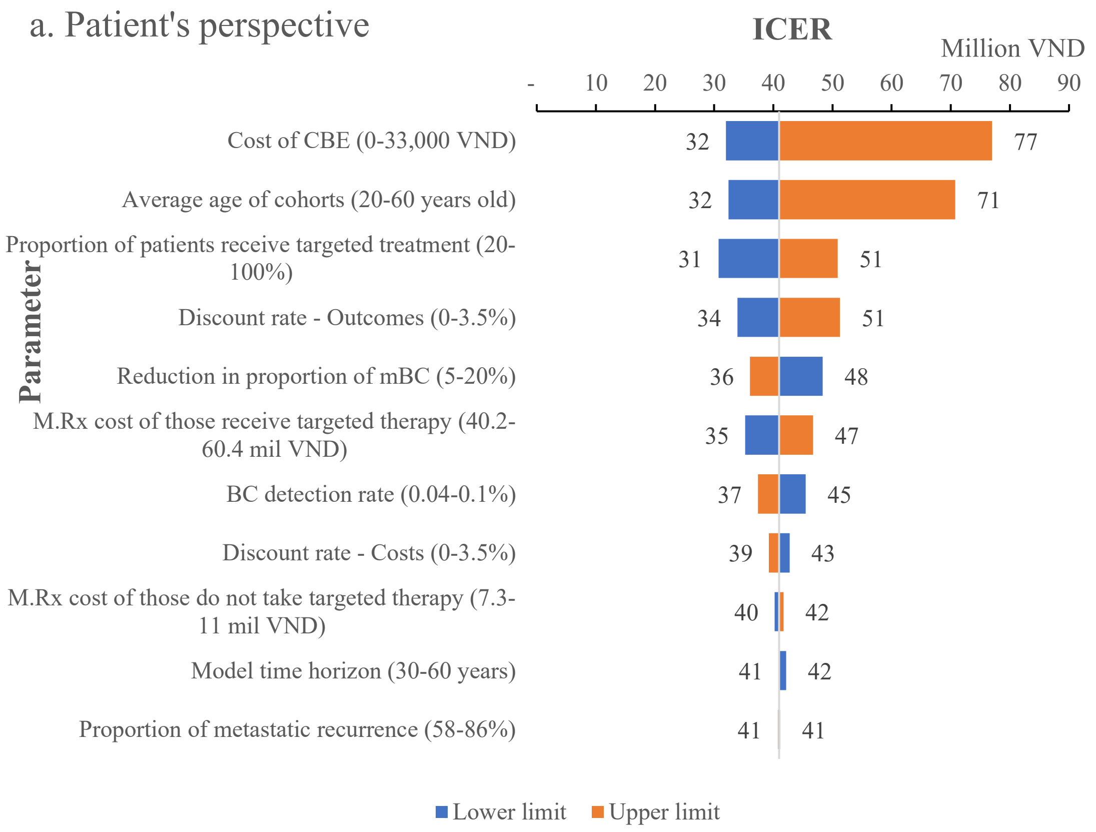

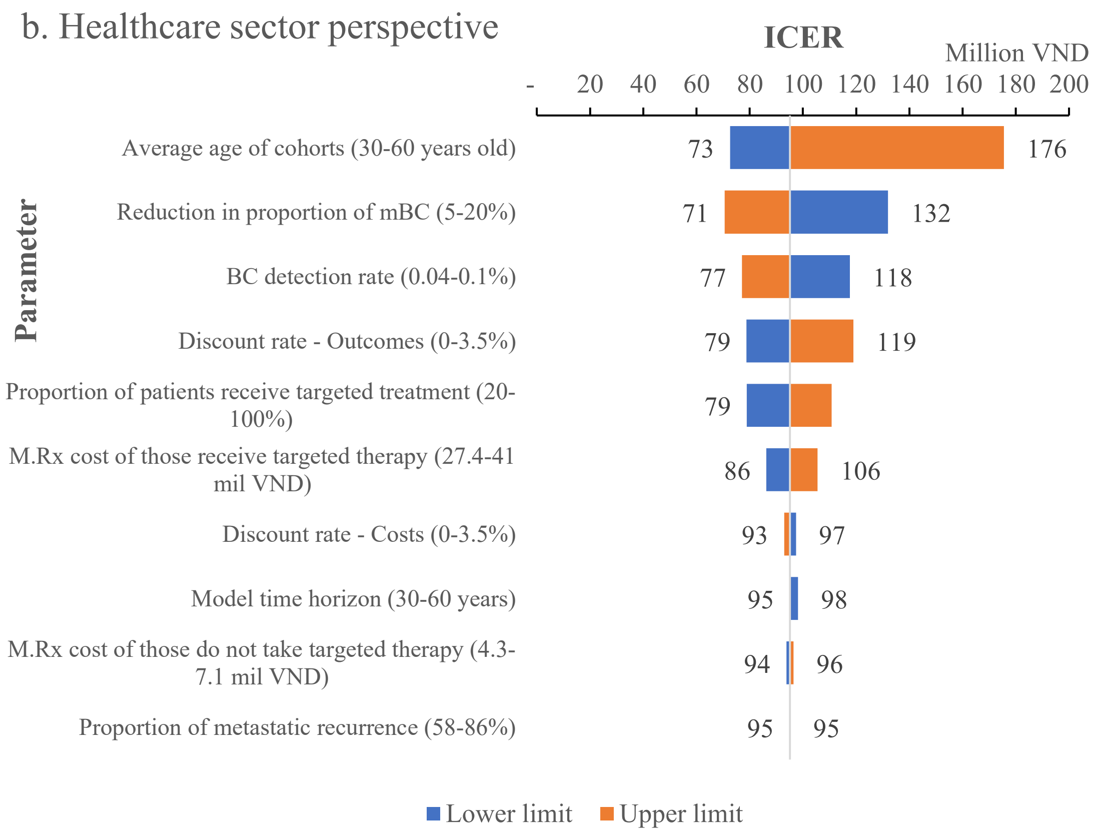

Supplement: Supplementary file 1 — Supplementary file1 (PDF 404 KB) [file 12282_2022_1398_MOESM1_ESM.pdf]
